# Mutualistic coupling of vocabulary and non‐verbal reasoning in children with and without language disorder

**DOI:** 10.1111/desc.13208

**Published:** 2022-02-07

**Authors:** Sarah Griffiths, Rogier A. Kievit, Courtenay Norbury

**Affiliations:** ^1^ Psychology and Language Science University College London London UK; ^2^ Cognitive Neuroscience Department Donders Institute for Brain Cognition and Behavior Radboud University Medical Center Nijmegen The Netherlands; ^3^ Department of Special Needs Education University of Oslo Oslo Norway

**Keywords:** cognitive development, language, language disorder, mutualism, reasoning

## Abstract

Mutualism is a developmental theory that posits positive reciprocal relationships between distinct cognitive abilities during development. It predicts that abilities such as language and reasoning will influence each other's rates of growth. This may explain why children with Language Disorders also tend to have lower than average non‐verbal cognitive abilities, as poor language would limit the rate of growth of other cognitive skills. The current study tests whether language and non‐verbal reasoning show mutualistic coupling in children with and without language disorder using three waves of data from a longitudinal cohort study that over‐sampled children with poor language at school entry (*N* = 501, 7–13 years). Bivariate Latent Change Score models were used to determine whether early receptive vocabulary predicted change in non‐verbal reasoning and vice‐versa. Models that included mutualistic coupling parameters between vocabulary and non‐verbal reasoning showed superior fit to models without these parameters, replicating previous findings. Specifically, children with higher initial language abilities showed greater growth in non‐verbal ability and vice versa. Multi‐group models suggested that coupling between language and non‐verbal reasoning was equally strong in children with language disorder and those without. This indicates that language has downstream effects on other cognitive abilities, challenging the existence of selective language impairments. Future intervention studies should test whether improving language skills in children with language disorder has positive impacts on other cognitive abilities (and vice versa), and low non‐verbal IQ should not be a barrier to accessing such intervention.

## 1 INTRODUCTION

In typical development, abilities across different cognitive domains are correlated, a phenomenon known as the “positive manifold” (Carroll, 1993; van der Maas et al., 2006). This phenomenon has often been attributed to the “g‐factor”, a single underlying latent variable that explains performance across domains. However, recent work has suggested that correlations between different cognitive domains, such as language and non‐verbal reasoning, may instead arise due to mutualism between domains throughout development (Kievit et al., 2017; van der Maas et al., 2006). According to mutualism theory, skill in one cognitive domain drives growth of skills in other domains and vice versa. This contrasts with a developmental “g‐factor” account that suggests that domains are correlated cross‐sectionally and longitudinally due to growth of a single underlying latent variable (Kievit et al., 2017). Studies of typically developing children have used (bivariate) Latent Change Score models (LCS; Ferrer & McArdle, 2010; Kievit et al., 2018; McArdle et al., 2000), to test mutualism theory by assessing the strength of coupling between baseline skill in one domain (e.g., vocabulary) and latent change in a skill in another other domain (e.g., non‐verbal reasoning). Kievit et al. (2019, 2017) found evidence for mutualistic coupling between vocabulary and non‐verbal reasoning in middle childhood (6–8 years; Kievit et al., 2019) and in adolescence (14–25 years; Kievit et al., 2017), such that individuals with the greater initial language skills showed greater growth in non‐verbal reasoning and individuals greater initial non‐verbal skills showed greater growth in language. These studies support mutualistic coupling between verbal and non‐verbal skills in typical development. However, it is less clear whether mutualistic coupling occurs between domains when skills in one or more domain are impaired due to a neurodevelopmental disorder (Ferrer et al., 2010).

Language disorders are a common feature of many neurodevelopmental conditions, while Developmental Language Disorder (DLD) is the internationally agreed diagnostic term for children with persistent language disorder without a known biomedical cause (e.g., autism, down syndrome, hearing loss; Bishop et al., 2017). DLD subsumes Specific Language Impairment (SLI), a diagnostic label that applied only to children with impaired language but relatively unimpaired non‐verbal cognitive skills. In other words, the SLI required a discrepancy between language and other cognitive abilities. A legacy of SLI is the requirement by some services that children have non‐verbal IQ scores within the normal range in order to receive specialist services and language intervention (Dockrell et al., 2006). Mutualism challenges this perspective, as early language disorder is likely to have downstream adverse effects on other cognitive domains early in development, and therefore such selective deficits should be rare (Reilly et al., 2014). In line with the predictions of mutualism theory, longitudinal studies of children with a history of SLI suggest that a large proportion of these children experience decreases in age‐normed non‐verbal IQ over time, such that they no longer meet strict SLI diagnostic criteria by adolescence (Botting, 2005; Conti‐Ramsden et al., 2012). Furthermore, many children with language disorder at school entry also have below average non‐verbal IQ scores that would exclude them from an SLI diagnosis (Norbury et al., 2016). These findings are consistent with the prediction from mutualism theory that language impairments early in development would reduce growth in non‐verbal reasoning, leading non‐verbal reasoning to become poorer (relative to peers) with increasing age (Peng & Kievit, 2020).

However, in other developmental conditions there is some evidence for weaker or even absent coupling between a primary impaired domain and other cognitive domains. For example, Ferrer et al. (2010) compared coupling between full‐scale IQ (including vocabulary and non‐verbal reasoning) and reading ability in typical readers and poor readers using multi‐group LCS models. In typical readers, full scale IQ and reading ability showed mutualistic coupling effects. However, in poor readers, there was no coupling from reading to full scale IQ, and coupling from full scale IQ to reading was weaker than in typical readers. The authors suggest that reduced coupling from general cognitive skills to reading may explain why dyslexia can occur in children that do not have other cognitive deficits (Ferrer et al., 2010).

**Research Highlights**

- Recent evidence has shown that language and non‐verbal reasoning mutually influence each other's growth during typical development.
- We tested whether longitudinal interactions between language and non‐verbal reasoning differed between children with and without language disorder (*N* = 501, 7–13 years).
- Multi‐group latent change score models found mutualistic positive relationships between language and reasoning in children with language disorders, as in typical development.
- Our results suggest that early language disorders have downstream effects on other cognitive abilities, providing evidence against selective language impairments.
- These findings highlight the need to measure downstream impacts of language interventions on other cognitive abilities as a strong test of mutualism theory.

Similarly, Quinn et al. (2020) investigated mutualistic coupling of vocabulary and reading comprehension, using separate LCS models for children with and without school‐identified learning disability (based on recorded eligibility for special educational need support). A model with coupling was a better fit than one without coupling for the typically developing group. However, for children with learning disabilities both models fit the data equally well. Parsimony led the authors to conclude that mutualistic coupling is disrupted in children with learning disabilities. However, the lack of evidence for better fit of the mutualism model may have been due to lower statistical power to detect an improvement in fit in the learning‐disabled group, given that this group was small relative to the typical group (learning disabled; *n* = 627, typical development; *n* = 14,146). Building separate models for the two groups, rather than implementing a multi‐group model (Ferrer et al., 2010) means that the strengths of coupling parameters were not directly compared between groups, limiting the ability to draw strong conclusions about group differences.

The aim of the current study is to test whether there is mutualistic coupling between receptive vocabulary and non‐verbal reasoning in a large and diverse population cohort, and if so, whether the strength of either coupling parameter differs between children with language disorder and those with typical language development. We first attempt to replicate findings from Kievit et al. (2019) using the same analysis code, by testing whether the mutualism model fits data better than models with coupling in only on direction, in our more cognitively diverse sample. We then run a multi‐group LCS model comparing children with and without language disorder, to test whether mutualistic relationships between language and non‐verbal reasoning differ between these groups.

## 2 METHODS

### 2.1 Sample

Data are from the Surrey Communication and Language in Education Study (SCALES; Norbury et al., 2016); a prospective, population derived cohort study tracking language development and associated outcomes in children with and without language disorder. Participants were screened for language difficulties at school entry using a short teacher‐report version of the Children's Communication Checklist, which is designed to identify communication problems, with higher scores indicating poorer language (CCC‐S; unpublished) based on the Children's Communication Checklist 2 (Bishop, 2003). A stratified random sample of 636 monolingual children attending mainstream infant's schools were selected for in‐depth assessments of language and cognition. Children identified as having low language (scoring > 1SD above the mean on the CCC‐S for their gender and season of birth) in the screening phase, were oversampled for in depth assessment. Performance on six standardized language tests in Year 1 was used to determine whether participants met diagnostic criteria for language disorder (for details see; Norbury et al., 2016).

The current analysis uses data from receptive vocabulary and non‐verbal reasoning assessments conducted when the children were in Year 3, Year 6 and Year 8. All assessments were conducted in‐person by trained researchers and took place at the child's school. These measures were completed by 501 children (260 male; mean age: 7.94 years, range: 7.08–9.25) in Year 3, 384 children (196 male; mean age: 11.16 years, range: 10.42–12.00 years) in Year 6 and 196 children (106 male; mean age 12.73 years, range: 12.08–13.83) in Year 8. The mean time between test visits in Year 3 and Year 6 was 39 months (range 22–51) and the mean time between test visits in Year 6 and Year 8 was 19 months (range 10–25). Attrition in Year 8 was greater than expected due to testing being halted by Covid‐19 pandemic school closures. Figure S1 in the supplementary material provides a consort diagram showing flow of participants through the study. There were no significant differences between children seen in Year 8 and those not seen in Year 8, or between those seen in Year 6 and not seen in Year 6 in terms of sex, language disorder status, receptive vocabulary or non‐verbal reasoning scores in Year 3 (See Table S1 in the supplementary material).

### 2.2 Measures

Receptive vocabulary was measured using the Receptive One Word Picture Vocabulary Test (ROWPVT; Brownell, 2001). In this test, participants are presented with single spoken words and have to select the corresponding picture from a choice of four. In Year 3 and Year 6, the pictures were presented in a stimulus book and responses were recorded manually by the researcher. In Year 8, the pictures were presented on a laptop via the internet platform Gorilla during the testing session at school, and the participant entered their response by clicking a button on the screen. In all sessions, starting and stopping rules were followed according to the test manual. The starting item is selected according to the child's age, a basal score is established by eight consecutive correct answers, and ceiling was established by six or more errors in eight consecutive trials. The maximum score is 190. According to the manual, the Cronbach's alpha is 0.95–0.97 and the test‐retest reliability coefficient for raw scores is 0.97.

Non‐verbal reasoning was measured using the Block Design task from Wechsler Intelligence Scale for Children‐IV (Wechsler, 2003). In this task, participants recreate patterns from pictures using colored cubes that have two red sides, two white sides and two sides that are half red and half white. Basal score is established by two consecutive correct answers and ceiling is established by three consecutive errors. Points are given for both accuracy and speed, with a max score of 68 points. The manual reported reliability of 0.86.

### 2.3 Analysis

**FIGURE 1:**
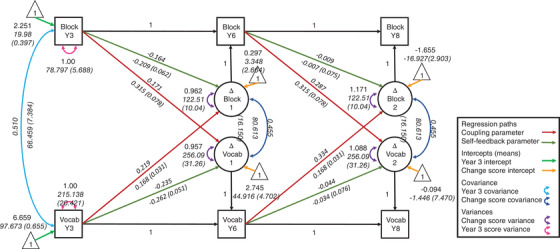
Bivariate latent change score model with mutualistic coupling parameters between block design and receptive vocabulary for the whole sample. Standardized estimates are in roman font and unstandardized estimates (and standard errors) are in italics

Structural Equation Modelling was conducted with Lavaan (version 0.6‐3) in R version (3.5.3) using robust full information maximum‐likelihood estimation to account for missing data and deviations from normality. Prior to analysis, we rescaled Year 6 and Year 8 scores to account for the variation in intertest interval between individuals^1^ (Ferrer & McArdle, 2004; Kievit et al., 2017). We used bivariate LCS models (Ferrer & McArdle, 2010; Kievit et al., 2018) to examine dynamic developmental relationships between receptive vocabulary and non‐verbal reasoning (Figure 1). LCS models use change in scores from T1 to T2 as perfect indicators of latent change (circles in Figure 1). This is achieved by setting the auto‐regression parameter weights to 1, setting the mean and variances of T2 scores to 0 and setting the factor loading of T2 score on latent change to 1. The intercept (yellow arrows in Figure 1) and variance (purple arrows in Figure 1) of the latent change scores indicate the average amount of change from T1 to T2 and variability in change from T1 to T2, respectively, conditional on the coupling and self‐feedback effects. Self‐feedback regression parameters (green arrows in Figure 1) from T1 score to latent change capture the association between score at T1 and change from T1 to T2. Positive self‐feedback parameters indicate that those that scored highest at T1 make the most progress, whereas negative self‐feedback parameters indicate that those that scored lowest at T1 make the most progress. Bivariate latent change score models, allow us to test hypotheses about mutualistic relationships between the two variables. Specifically, we test whether T1 scores for one variable predict *change* in scores from T1 to T2 in the other variable (red arrows in Figure 1). We can also test whether change in the two variables are correlated (dark blue arrows in Figure 1).

We first built a 3‐wave bivariate LCS model for the whole sample using R code made available by Kievit et al (2019, 2017). As in Kievit et al (2017), we used raw scores as indicator variables, but extended the model to include 3‐waves of data as in Kievit et al (2019). Equality constraints were imposed on the same parameters across waves where possible but estimated freely where necessary. Equality constraints specified that the latent change intercept between Year 6 and Year 8 was 0.67 times the latent change intercept between Year 3 and Year 6 to account for the unequal time interval. All observations were included, and missing data was assumed to be missing at random and dealt with using Full Information Maximum Likelihood, which yields unbiased parameter estimates if MAR conditions are met (Enders & Bandalos, 2001). Model fit was assessed using the chi‐square test, the root‐mean‐square error of approximation (RMSEA; acceptable fit: < 0.08, good fit: < 0.05), the comparative fit index (CFI; acceptable fit: 0.95–0.97, good fit: > 0.97), and the standardized root‐mean‐square residual (SRMR; acceptable fit: 0.05–0.10, good fit: < 0.05; Schermelleh‐Engel et al., 2003). We compared the mutualism model to a model without coupling parameters by comparing overall model fit using the likelihood ratio test.

We next sought to determine whether there were differences in developmental relationships of receptive vocabulary and non‐verbal reasoning between children with and without language disorder. We first ran multi‐group univariate LCS models for vocabulary and non‐verbal reasoning separately to determine whether groups differed in mean scores in Year 3, variance in scores in Year 3, rate of change and variance in change. These parameters were estimated freely for each group in the initial model. We then constrained each parameter to equality between groups, and tested whether this led to a decrease in model fit using a chi‐squared test. If constraining the parameters leads to a decrease in model fit this provides evidence that the parameter differs between groups (Kievit et al., 2018). We then combined the two grouped univariate models to create a bivariate LCS model with coupling parameters, and covariance between Year 3 scores on the vocabulary and block design, free to vary between groups. Parameters that were shown to differ between groups in the univariate models were left free in the bivariate model, while all other parameters were constrained to equality. Again, we compared this model with free coupling and covariance parameters to a model with each parameter constrained, to test whether the strength of mutualistic coupling and covariance between vocabulary and non‐verbal reasoning, differs between children with and without LD.

## 3 RESULTS

**FIGURE 2:**
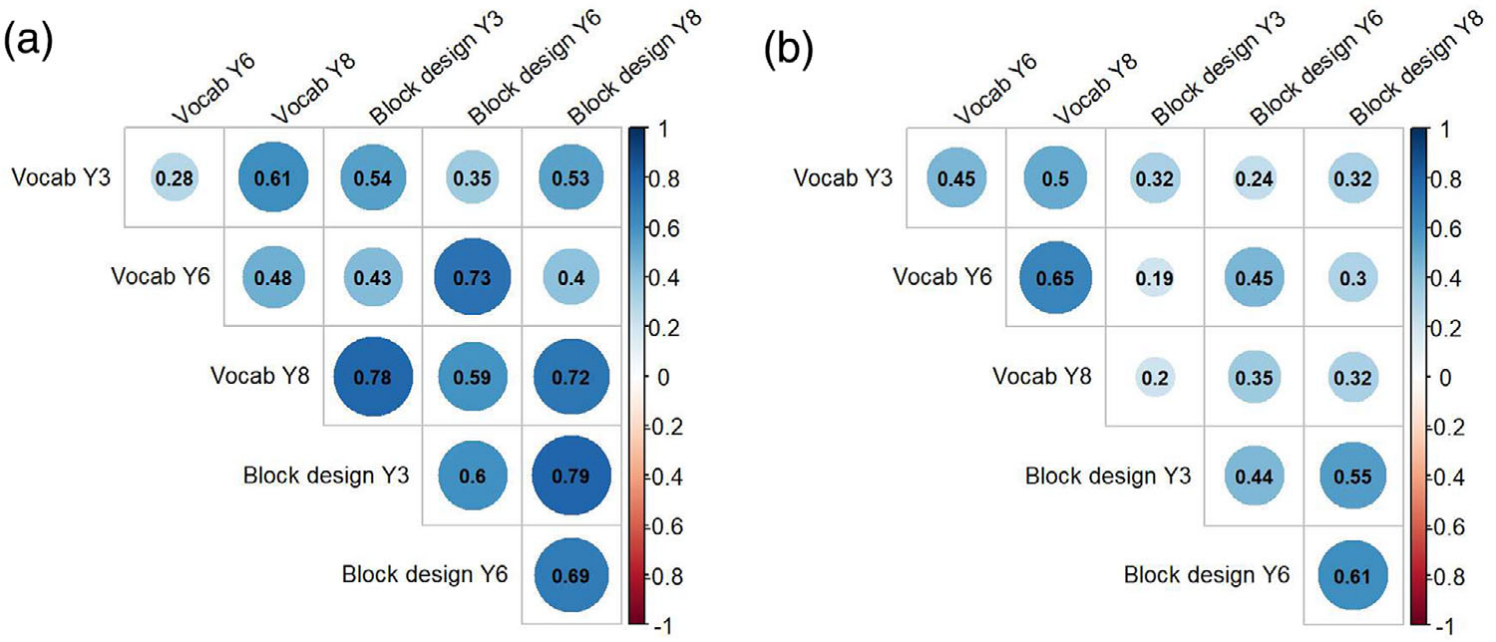
Correlation coefficients for each combination of variables at each wave for (a) children with LD and (b) children with typical language

Mean raw scores for receptive vocabulary and block design at each time point for the whole sample and each language group are reported in Table 1. Correlations between receptive vocabulary and block design measurements at each time point are presented in Figure 2. Standardized scores at each time point are presented in Figure S2 in supplementary material.

**TABLE 1:** Descriptive statistics for receptive vocabulary and block design measures shown for the whole sample and the typical and LD group separately

	Whole sample			Typical language group			LD group		
	N	Mean	SD	N	Mean	SD	N	Mean	SD
Receptive vocabulary Y3	501	97.67	14.68	372	102.55	10.88	129	83.60	15.17
Receptive vocabulary Y6	384	123.55	20.69	281	128.97	17.99	103	108.75	20.41
Receptive vocabulary Y8	193	128.44	19.56	138	135.45	14.87	55	110.87	18.96
Block design Y3	498	20.02	8.86	371	21.87	8.21	127	14.64	8.51
Block design Y6	382	35.93	13.69	281	38.45	12.36	101	28.91	14.79
Block design Y8	195	38.6	14.57	140	42.47	12.43	55	28.76	15.08

Two exceptions to cross‐wave equality constraints were necessary: (1) Model comparison suggested that the change intercepts need to be freely estimated, as there was a greater amount of (conditional) growth per year between year 3 and 6 compared to between year 6 and 8, and (2) Model comparison suggested that the self‐feedback parameters needed to be freely estimated, as they were stronger between year 3 and 6 than between year 6 and 8. Doing so, the mutualism model for the whole sample (Figure 1) had good fit; X^2^ (5) = 7.98, *p* = 0.157, RMSEA = 0.034, 90% Confidence Interval (CI) = [0.000, 0.077], CFI = 0.997, SRMR = 0.024. Coupling parameters (red arrows in Figure 1) were significant both from vocabulary to block design (year 3 to year 6; r = 0.22 95% CI [0.05, 0.39], year 6 to year 8; r = 0.33, 95% CI [0.06, 0.61]) and from block design to vocabulary (year 3 to year 6; r = 0.17 95% CI [0.07, 0.37], year 6 to year 8; r = 0.29 95% CI [0.12, 0.45]), replicating Kievit et al. (2017) and Kievit et al. (2019). Self‐feedback parameters were negative and significant for growth from Year 3 to Year 6 but not significant for growth from Year 6 to Year 8. This indicates that the higher a child's score in one domain in Year 3 the less conditional growth they will experience between Year 3 and Year 6 in the same domain. Removing coupling parameters from vocabulary to block design lead to a reduction in model fit ∆X^2^ (1) = 38.12, *p* < 0.001, as did removing coupling parameters from block design to vocabulary ∆X^2^ (1) = 21.93, *p* < 0.001. This suggests that the model with coupling between vocabulary and reasoning in both directions is more consistent with the data than a model without these coupling effects.

We next fit multi‐group univariate LCS model for vocabulary and block design. In both models, Year 3 intercept, change intercepts, and change variance were freely estimated for each group. For vocabulary, the model fit the data well: vocab: X^2^ (5) = 5.33, *p* = 0.377, RMSEA = 0.016, 90% Confidence Interval (CI) = [0.000, 0.076], CFI = 0.997, SRMR = 0.043. However, for block design there was some evidence of misfit; X^2^ (5) = 13.38, *p* = 0.020, RMSEA = 0.082, 90% Confidence Interval (CI) = [0.031, 0.135], CFI = 0.960, SRMR = 0.092. Allowing the self‐feedback parameters to differ between groups lead to a significant improvement in model fit; ∆X^2^(2) = 9.98, *p* = 0.007, and this model fit the data well; X^2^ (3) = 3.99, *p* = 0.262, RMSEA = 0.036, 90% Confidence Interval (CI) = [0.000, 0.115], CFI = 1.00, SRMR = .036. Self‐feedback parameters were negative and significant from Year 3 to 6 in the typical language group but not LD group, meaning greater reasoning ability in Year 3 predicted less conditional change from Year 3 to Year 6 for children with typical language but not children with language disorder.

Constraining Year 3 intercepts to equality across language groups led to a decrease in model fit for both vocabulary; ∆X^2^ (1) = 68.79, *p* < 0.001, and block design; ∆X^2^ (1) = 53.38, *p* < 0.001, as children with LD start with lower receptive vocabulary and block design scores in Year 3 than their peers with typical language. Constraining variance in Year 3 scores to equality between groups also led to a decrease in fit for vocabulary; ∆X^2^ (1) = 22.39, *p* < 0.001, but not block design ∆X^2^ (1) = 0.26, *p* = 0.608; due to greater variability in vocabulary scores in the LD group compared to the typical language group in Year 3. Constraining the intercept for change from Year 3 to Year 6 between language groups lead to a decrease in model fit for both vocabulary ∆X^2^ (1) = 6.80, *p* = 0.009; and block design; ∆X^2^ (1) = 17.29, *p* < 0.001, with children with LD having smaller conditional change estimates on both measures between Year 3 and Year 6. In contrast, constraining the intercept for change from Year 6 to Year 8 did not reduce model fit for vocabulary, ∆X^2^ (1) = 0.95, *p* = 0.33, or block design ∆X^2^ (1) = 0.004, *p* = 0.95. Similarly, constraining change score variances between groups did not lead to a drop in model fit for vocabulary; ∆X^2^ (1) = 0.01, *p* = 0.93 or block design; ∆X^2^ (1) = 0.31, *p* = 0.58, suggesting that the degree of individual differences in change of vocabulary and block design is similar for children with and without LD.

**FIGURE 3:**
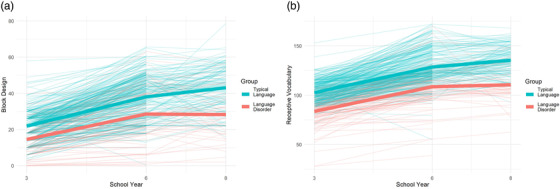
Growth in scores for (a) block design and (b) receptive vocabulary for children with typical language and those with language disorder. The thick line shows the model predicted score for each group for each measurement occasion

**FIGURE 4:**
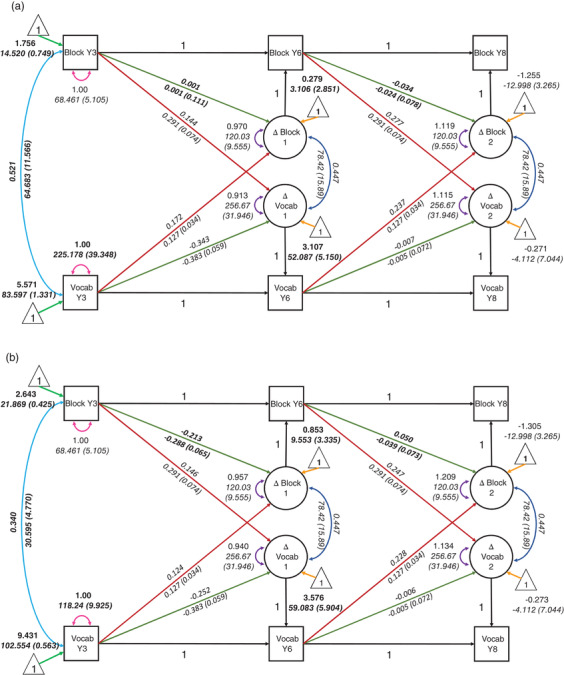
Multi‐group bivariate Latent Change Score model with mutualistic coupling parameters between block design and receptive vocabulary. Standardized estimates are in roman font and unstandardized estimates (and standard errors) are in italics. Panel (a) shows estimates for children that met the criteria for LD and panel (b) shows estimates for children with typical language

The grouped bivariate LCS model with coupling parameters, and covariance between scores in Year 3, freely estimated for each group, fit the data well: X^2^(22) = 26.05, *p* = 0.249, RMSEA = 0.027, 90% Confidence Interval (CI) = [0.000, 0.060], CFI = 0.993, SRMR = 0.057. Constraining the covariance between vocabulary and block design in Year 3 to be equal between groups led to a significant drop in model fit; ∆X^2^ (1) = 11.17, *p* < 0.001, with greater covariance between vocabulary and block design observed for children with LD relative to children with typical language. Constraining the coupling parameters from vocabulary to block design between groups did not lead to a drop in model fit; ∆X^2^ (1) = 0.03, *p* = 0.858 nor did constraining the coupling parameter from block design to vocabulary; ∆X^2^ (1) = 0.075, *p* = 0.784. The final model with coupling parameters constrained to equality but covariance estimated freely for each group fit well X^2^(24) = 30.10, *p* = 0.182, RMSEA = 0.032, 90% Confidence Interval (CI) = [0.000, 0.064], CFI = 0.991, SRMR = 0.055. Figure 3. shows the model predicted scores for each group at each time point with the raw scores for each participant. Parameter estimates for each group can be found in Figure 4.

## 4 DISCUSSION

The current study tested mutualistic coupling in growth of receptive vocabulary and non‐verbal reasoning in late childhood, in children with typical language development and those that met the criteria for language disorder. We used bivariate LCS models to test coupling across three waves of measurements of receptive vocabulary and block design from age 7 to age 13. Models with mutualistic coupling parameters from vocabulary to non‐verbal reasoning and from non‐verbal reasoning to vocabulary showed superior fit to models that included coupling in one direction only, providing support for a mutualistic relationship between verbal and non‐verbal skill development. Coupling parameters were uniformly positive and ranged from moderate (r = 0.13) to large (r = 0.28) in magnitude (Gignac & Szodorai, 2016). In other words, individuals with higher scores in vocabulary showed greater gains in non‐verbal reasoning and vice versa.

Our finding of mutualistic coupling between receptive vocabulary and non‐verbal reasoning replicate those of Kievit et al. (2017) and Kievit et al. (2019) in an age range that falls between those included in these two previous studies. Despite using a different measure of non‐verbal reasoning (block design instead of matrix reasoning) and having a more cognitively diverse sample, our estimates for coupling parameters are strikingly similar. Mutualism theory predicts that coupling will become weaker with age due to increasing alignment of abilities (Kievit et al., 2017). Our study age range of 7–13 years lies between that of Kievit et al (2019); aged 6–8 years, and Kievit et al (2017); aged 14–25 years, and our parameter estimates in the ungrouped model also lie between parameter estimates the two previous studies in their strength. For the coupling parameter from non‐verbal reasoning to vocabulary our standardized estimates were 0.19 for wave 1–2 and 0.28 for wave 2–3, compared to 0.22 for wave 1–2 and 0.28 for wave 2–3 for the previous younger sample (Kievit et al, 2019); and 0.16 for the previous older sample (Kievit et al, 2017). For the coupling parameter from vocabulary to non‐verbal reasoning our standardized estimates were 0.22 for wave 1–2 and 0.32 for wave 2–3 compared to 0.33 for wave 1–2 and 0.45 for wave 2–3 for the previous younger sample and 0.20 for the previous older sample. Our findings therefore provide further evidence for the mutualism theory of cognitive development.

We found no evidence that the strength of coupling differed between children with language disorder and children with typical language, suggesting that this mechanism is intact even in children with neurodevelopmental disorders characterized by low initial levels of language. This provides important evidence against the existence of selective language impairments. With intact mutualism, even if a selective language disorder existed very early in development, we would expect low initial level of language to reduce growth in other cognitive domains, resulting in multiple deficits as abilities become increasingly correlated. This prediction is in line with previous findings from the SCALES cohort used in this study that only 14% of children with DLD met criteria for Specific Language Impairment, involving a discrepancy between verbal and non‐verbal ability (Norbury et al., 2016), and evidence that age‐normed non‐verbal IQ scores decrease over time in children that initially show poor language skills relative to their non‐verbal skills (Botting, 2005; Conti‐Ramsden et al., 2012).

Mutualistic coupling in children with language disorder also has important implications for understanding language growth in children with language disorder. With intact mutualism in children with language disorder, we would expect coupling to lead to persistent, but not worsening deficits in language, as domains support growth in each other. Previous analyses, including those with data from the SCALES cohort used in this study, have found that children with language disorder show parallel rates of growth in language when compared to children with typical language (Bornstein et al., 2016; Norbury et al., 2017). Mutualism theory offers a plausible explanation for stable language growth trajectories in children with language disorder (Bornstein et al., 2016; Norbury et al., 2017).

Our finding of intact mutualistic coupling in children with a developmental disorder contrast with previous findings of weaker coupling between reading and other cognitive domains in dyslexia. Ferrer et al. (2010) identified a group of persistently poor readers, and a group of compensated poor readers. Compensated readers had higher initial cognitive ability and showed stronger coupling from cognitive skills to reading. Weaker coupling was thought to explain the slower growth in reading in the persistently poor readers. The difference between our findings in children with language disorder and previous findings in children with dyslexia, may be due to the fact that children learn oral language through regular incidental exposure, while reading is learnt through explicit instruction and practice. Ferrer et al. (2010) suggest that uncoupling of reading and cognitive skills may be due to dyslexic readers avoiding practicing reading. Dyslexic children may therefore have reduced opportunity to use their cognitive ability to improve their reading outside of the classroom. In contrast, children with language disorder cannot avoid oral language, perhaps resulting them benefiting from their existing reasoning abilities, even if they are weak for their age.

The strengths of our study include the use of a relatively large epidemiological cohort that included children with the full range of language and cognitive abilities, use of scores from standardized cognitive tests and modelling change across three‐time points. However, there are some limitations. First, we used single indicator variables to measure receptive vocabulary and non‐verbal reasoning. Both measures are valid measures with good reliability in this age range. They are the same (or very similar in the case of non‐verbal reasoning) measures that have been used to demonstrate mutualism in typically developing children, allowing us to directly compare our results (Kievit et al., 2019; Kievit et al., 2017). None‐the‐less, the use of multiple indicators to construct latent variables at each time‐points would have reduced measurement error. Second, we only looked at a single ability in two cognitive domains but mutualism theory would predict coupling between abilities in all domains and it is possible that there is disrupted coupling in children with language disorder between different aspects of language and other cognitive skills that we did not measure. Future research should explore whether mutualistic coupling is intact between other languages abilities, such as grammar, and reasoning skills in language disorders. Finally, in the current study children with low language ability were oversampled allowing us to test whether having language disorder disrupts the coupling between language and reasoning. A related question is whether having intellectual disability disrupts mutualistic coupling between language and reasoning. Based on the current data we would hypothesis that it would not, given many children with language disorder also have lower than average non‐verbal ability, and we did not find any evidence for disrupted coupling in this group. However, this is a question for future research.

Our findings have some important implications for practice. One result of the traditional non‐verbal IQ exclusion criteria for SLI diagnosis is that in some regions, children with low non‐verbal IQ have been unable to access language interventions (Dockrell et al., 2006) and are rarely included in clinical or educational trials. Our findings raise the possibility that targeting these children's language could not only improve their language but could also improve other cognitive skills. Our findings therefore provide further empirical support for recommendations by CATALISE (Bishop et al., 2017) and the American Speech‐Language‐Hearing Association (American Speech‐Language‐Hearing Association, 2003) that children with low non‐verbal IQ should not be excluded from speech and language interventions.

## CONFLICT ON INTERESTS

The authors have no conflicts of interest

## ETHICS STATEMENT

Consent procedures and study protocol were developed in consultation with Surrey County Council and approved by the Royal Holloway Ethics Committee and the University College London (UCL) Research Ethics Committee 9733/002)

## Supporting information

Supporting information.

## Data Availability

Analysis code and partial data are available at the Open Science Framework: osf.io/pg689. Twelve participants whose data is included in the reported analysis did not consent to having their data made openly available, so their data has been removed from the open dataset. The complete dataset can be requested from the authors.

## Footnotes

1 Raw scores were rescaled using the following formula;
$x_{ti}^{\prime }=\;x_{ti}+\;\left[\left(interval_{ti}^{\prime }-interval_{ti}\right)*\;\left(\frac{x_{ti}-\;x_{t-1i}^{\prime }}{interval_{ti}}\right)\right]$ Where $x_{ti}^{\prime }$ is the rescaled score at time *t* for person *i*, *x_ti_* is the original score, $interval_{ti}^{\prime }$ is the expected intertest interval (3 years from Year 3 to Year 6, 2 years from Year 6 to Year 8) and *interval_ti_* is the actual intertest interval.
