# Supplementary material for: Mutualistic coupling of vocabulary and non‐verbal reasoning in children with and without language disorder
Source: Dev Sci. 2022 Feb 7;25(3):e13208. doi: 10.1111/desc.13208 (PMC9132040; doi:10.1111/desc.13208)
Supplement: Supplementary file 1 — Supporting information. [file DESC-25-e13208-s001.docx]

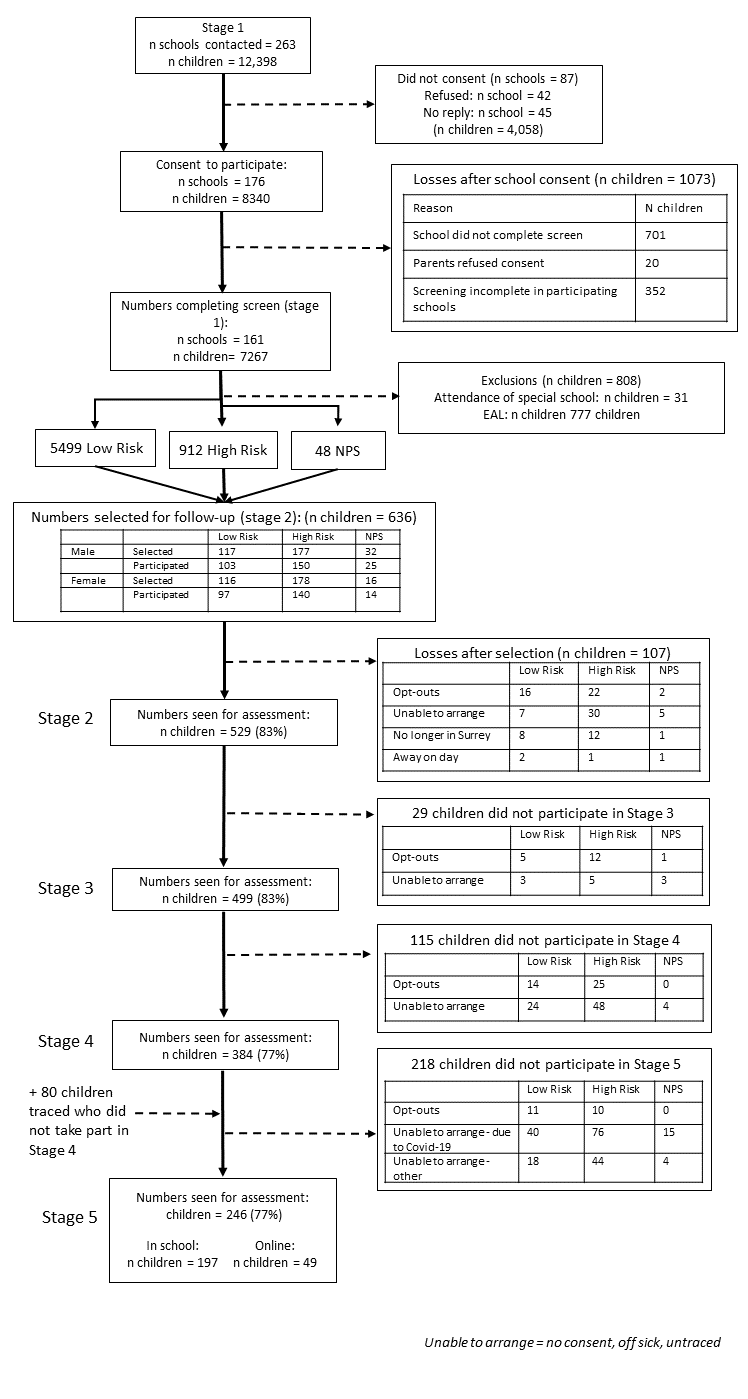


Figure S1. Consort diagram showing flow of participants through the study.

a)


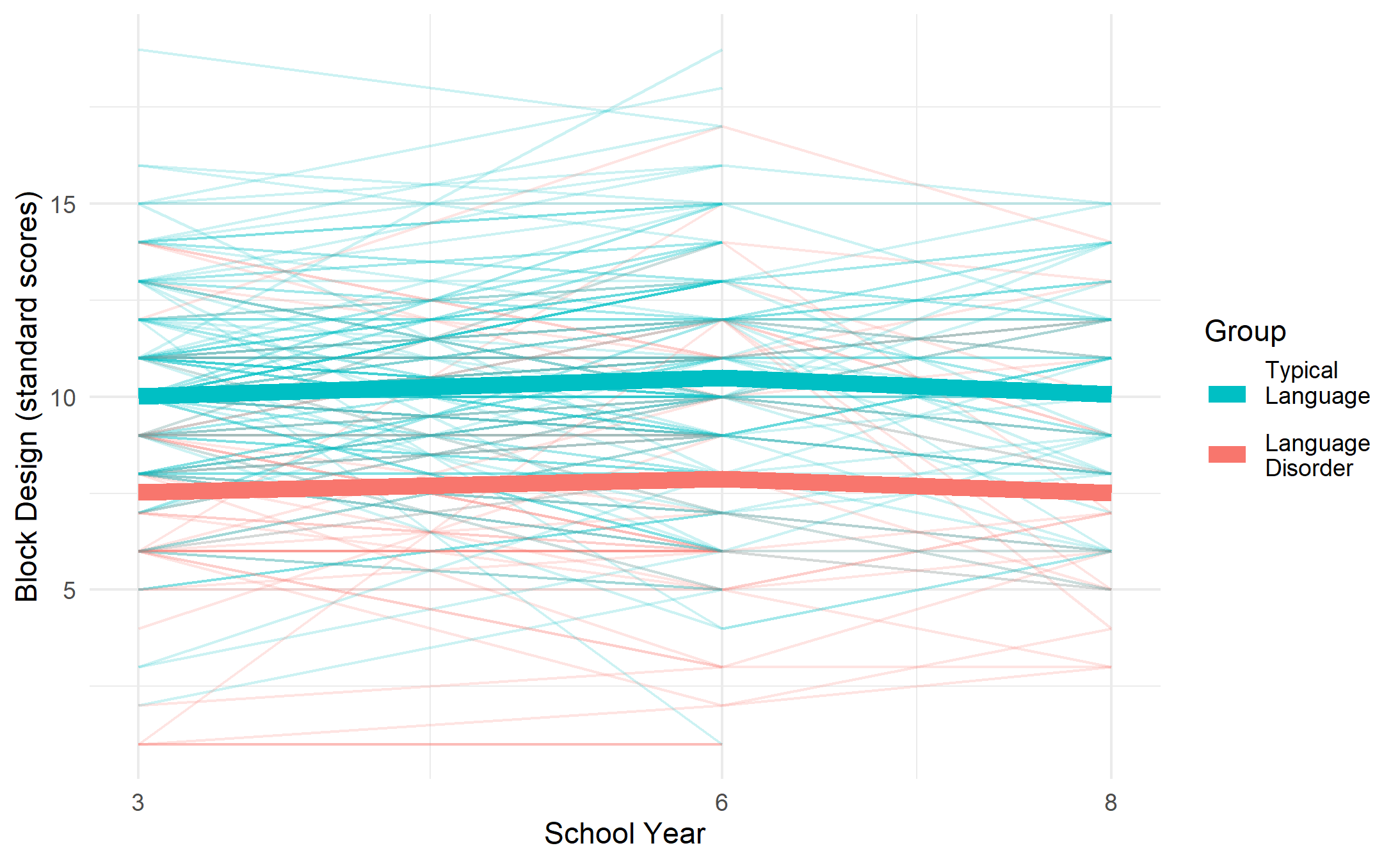


b)


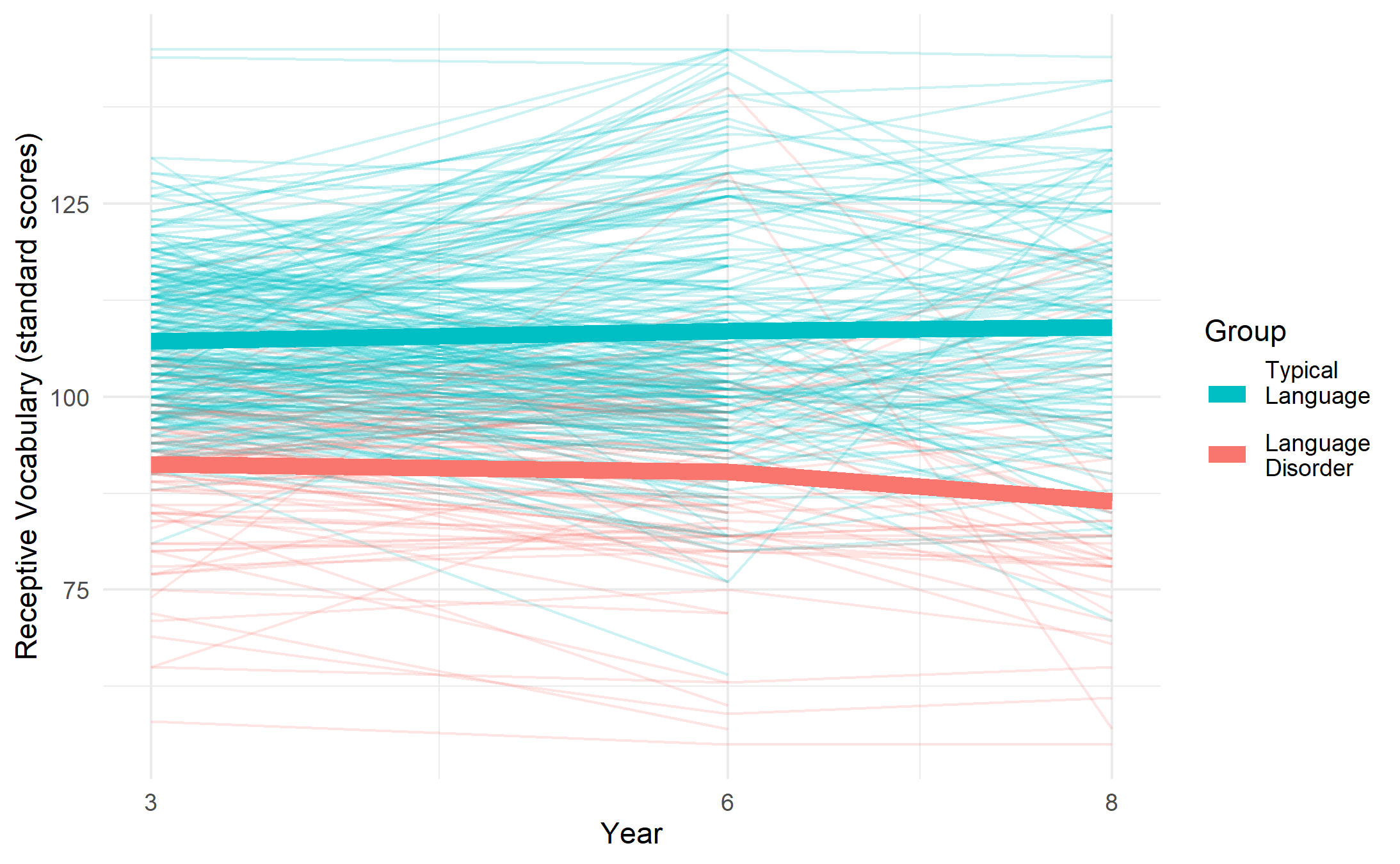
Figure S2. Standard scores for a) block design and b) receptive vocabulary for children with typical language and those with language disorder. The thick line shows the mean score for each group.

Table S1: Comparisons between children missing and not missing in Year 6 and Year 8.

|  | Seen Y6 | Missing Y6 |  | Seen Y8 | Missing Y8 |  |
| --- | --- | --- | --- | --- | --- | --- |
| N | 384 | 117 |  | 196 | 305 |  |
| Sex (N male) | 196 | 64 | **χ**^2^ (1) = 0.35,  *p* = .55 | 106 | 154 | **χ**^2^ (1) = 0.48,  *p* = .49 |
| Language Disorder (N) | 103 | 91 | **χ**^2^ (1) = 0.77,  *p* = .49 | 56 | 73 | **χ**^2^ (1) = 1.11,  *p* = .49 |
| ROWPVT Year 3 (Mean) | 97.98 | 96.65 | *t*(215) = 0.92, *p* = .38 | 96.71 | 98.29 | *t*(356) = 1.13,  *p* = .26 |
| Block design Year 3 (Mean) | 20.01 | 20.09 | *t*(208) = 1.83, *p* = .93 | 19.15 | 20.58 | *t*(447) = 1.81,  *p* = .07 |
